# New clinical developments in histone deacetylase inhibitors for epigenetic therapy of cancer

**DOI:** 10.1186/1756-8722-2-22

**Published:** 2009-06-01

**Authors:** Shundong Cang, Yuehua Ma, Delong Liu

**Affiliations:** 1 Division of Hematology/Oncology, New York Medical College, Valhalla, NY 10595, USA; 2 Henan Province People's Hospital, Zhengzhou, PR China

## Abstract

DNA methylation and histone acetylation are two well known epigenetic chromatin modifications. Epigenetic agents leading to DNA hypomethylation and histone hyperacetylation have been approved for treatment of hematological disorders. The first histone deacetylase inhibitor, vorinostat, has been licensed for cutaneous T cell lymphoma treatment. More than 11 new epigenetic agents are in various stages of clinical development for therapy of multiple cancer types. In this review we summarize novel histone deacetylase inhibitors and new regimens from clinical trials for epigenetic therapy of cancer.

## Introduction

DNA methylation and histone acetylation are two most studied epigenetic modifications, although ethyl, acetyl, phosphoryl, and other modifications of histones have been described [1,2]. Histone acetylation and methylation have been studied extensively in carcinogenesis [3]. Histone acetylases (HATs), histone deacetylases (HDACs), histone lysine methyltransferases (HMTs), and histone demethylases are key enzymes involved in epigenetic regulation and chromatin remodeling. Coordinated DNA methylation and histone modification play a key role in the control of gene expression [2]. Vorinostat (Zolinza, Merck) is the first HDAC inhibitor that has been licensed for clinical use [4-10]. More than 11 HDAC inhibitors are in clinical development. In this review we summarize novel HDAC inhibitors and new regimens from clinical trials for epigenetic therapy of cancer.

### Vorinostat (SAHA, Zolinza)

Eighteen enzymes from HDAC family have been identified in human [4,11-14]. Voriniostat (formerly, suberoylanilide hydroxamine, SAHA) is a pan-HDAC inhibitor [4,14]. SAHA has significant anti-cancer activity in a wide range of cancers [4,14-17]. Vorinostat (VOR) was studied in a phase II trial for patients with refractory cutaneous T-cell lymphoma (CTCL) [4,15-18]. 33 patients who have failed a median of 5 prior therapies were enrolled. Similar to other epigenetic agents, time to response to SAHA was 11.9 weeks. SAHA is well tolerated orally with common toxicities including fatigue, thrombocytopenia, nausea and diarrhea. 200 mg BID orally has the most favorable safety and efficacy profile [4,15-18]. A separate phase IIb trial which included 74 patients with refractory or persistent CTCL confirmed the activity of VOR [19,20]. 32% of the patients also had pruritus symptom relief. Pulmonary embolism was reported in 5% of the patients. VOR has been approved for treatment of refractory CTCL (Zolinza, Merck). Since then there have been more than 30 trials testing VOR in single agent or in combination (Table 1 and 2). In an analysis reported at the American society of Clinical Oncology (ASCO) 2008 annual meeting, 476 patients received VOR either as single agent or combined with another agent [21]. More than half of those patients had fatigue, nausea and diarrhea. Dose modifications were not required nevertheless in the majority of the patients.

**Table 1:** Vorinostat in single agent trials

HDACI	Disease	Dose & schedule	Phase	No. Pts	Outcome	Reference
Vorinostat	Various cancer	400 mg, oral	I	476	Tolerated, safe	[21]
Vorinostat	CTCL	200 mg Bid PO	IIb	74	CR: 16% PR: 67% SD: 16%	[20]
Vorinostat	GI cancer	300 mg Bid PO 3 days/w q21d	II	16	safe, PK results	[26]
Vorinostat	TCC	200 mg Bid PO	II	14	study closed	[36]
Vorinostat	Breast, colon, lung Cancer	200–400 mg Bid PO ×14q21d	II	16	SD: 50%	[22]
Vorinostat	MM	200–300 mg Bid PO × 5d/wq28d/200–400 mg Bid PO × 14d/wq21d	I	13	No response	[40]
Vorinostat	DLBCL	300 mg Bid PO 3 days/week	II	18	CR: 5.5% SD: 5.5%	[23]
Vorinostat	Gynecologic tumor	400 mg/d PO	II	27	Insufficient activity (PR: 3%)	[37]
Vorinostat	MDS, leukemia	100–300 mg Bid/TidPO ×14q21d	1	41	MTD:200 mg Bid	[24]
Vorinostat	Head neck cancer	400 mg qd PO	II	12	No response	[41]
Vorinostat	mesothelioma	NR	I	13	PR: 15%	[35]

CR: complete responses; PR: partial response; SD: stable disease. MTD: maximal tolerated dose; NR: not reported.

**Table 2:** Vorinostat in combination trials

HDACI	Other agent	Disease	Dosage	Phase	Pts No	Response	Reference
Vorinostat	capecitabine	Solid tumors	VOR:300–400 mg/d PO CAP:750–1000 mg bid PO	I	28	CR: 3% PR: 10% SD: 64%	[33]
Vorinostat	bevacizumab	kidney cancer	VOR:200 mg BID × 14 d PO BEV:15 mg/kg, q21div	I	8	SD: 42%	[27]
Vorinostat	bexarotene	CTCL	VOR:300–400 mgqdPOq28d BEX:150–300 mg/m^2^qdq28d	I	19	CR: 5% PR: 15% SD: 63%	[28]
Vorinostat	tamoxifen	breast cancer	VOR:400 mg/d ×21d PO T AM:20 mg/d PO	II	19	CR: 5% PR: 15%	[32]
							
Vorinostat	Gemcitabine Carboplatin/Cisplatin	NSCLC	VOR:300–400 mgqdPOx7d GEM:1000–1250 mg/m^2^, ivd3, 10 C BP:5.0AUC, iv/CDDP:75 mg/m^2^, iv d3	I	12	PR: 57% SD: 28%	[38]
Vorinostat	13-cis-retinoid acid	Pediatric CNS, solid tumors	VOR:180–230 mg/m^2 ^po qd 13cRA: 80 mg/m^2^po bid	I	13	MTD: VOR 180 mg/m^2^/d × 4/w 13cRA: 80 mg/m^2^bid	[39]
Vorinostat	Carboplatin Paclitaxel	solid tumors	VOR:400–600 mg/d PO q21d C BP:6.0 AUC, iv PTX:200 mg/m^2^, iv	I	28	PR: 44% SD: 28%	[34]
Vorinostat	bortezomib	MM	VOR:100–400 mg PO d4–11 VEL:1.3 mg/m^2 ^IV d1, 4, 8 and 11	I	23	MTD: VOR 400 mg d 4–11;VEL1.3 mg/m^2^d1, 4, 8, 11.	[25]

VEL: bortezomib; CR: complete responses; PR: partial response; SD: stable disease. MTD: maximal tolerated dose;

In a multicenter phase II single agent study, 16 patients with breast, colon and lung cancers received VOR at doses of 200, 300, and 400 mg BID for 14 days out of every 3 weeks. Disease stabilization was observed in half of the patients, but there were no confirmed responses [22].

In a single agent phase I study of VOR for patients with recurrent diffuse large B-cell lymphoma, 2 of the 18 enrolled patients had response, the other 16 had disease progression [23]. 300 mg BID 3 days/week was well tolerated but with limited activity.

Single agent VOR was also investigated in a phase I study for patients with leukemia and myelodysplasia (MDS) [24]. Thirty one of the 41 patients enrolled had acute myeloid leukemia (AML). VOR was given twice or thrice daily for 14 days with dose range of 100–300 mg in a 21-day cycle. The Maximal tolerated dose (MTD) was 200 mg BID. Seven patients had hematological improvement, including 4 AML with complete responses. Increased histone acetylation was seen at all dose levels.

VOR was combined with bortezomib in a phase I trial for patients with relapsed or refractory multiple myeloma (MM) [25]. The dose limiting toxicity (DLT) was QT prolongation and fatigue. The MTD was VOR 400 mg on days 4–11 and bortezomib 1.3 mg/m^2 ^on day 1, 4, 8, 11.

In a phase I trial for Japanese patients with gastrointestinal cancer, DLT of single agent VOR was grade 4 thrombocytopenia [26]. In this group of 16 Japanese patients, 300 mg BID for 3 consecutive days followed by a 4-day rest each week was the tolerable regimen.

In a small phase I study of patients with stage IV renal cell carcinoma, VOR 200 mg BID × 14 days was combined with bevacizumab 15 mg/kg on a 21-day cycle [27]. Eight patients were enrolled. Severe thrombocytopenia was the DLT at the above dosing schedules (MTD). Phase II study is underway.

In a preliminary report of a phase I dose-escalating trial of VOR plus bexarotene for advanced CTCL, 19 patients were included [28]. MTD was not reached yet. 1 patient had a CR, 3 had a PR, and 12 had stable disease.

HDAC inhibitors have been reported to restore hormone sensitivity of estrogen and progesterone receptors [29-31]. VOR 400 mg daily × 3 weeks was combined with daily tamoxifen in a 4-week cycle for patients with hormone-refractory breast cancer [32]. 17 of the 19 enrolled patients were evaluable. Four patients had objective response, one of them was in CR. H3 and H4 histone acetylation was seen at day 8. These findings imply that VOR may restore hormone sensitivity in hormone-refractory breast cancer patients.

VOR was investigated in combination with capecitabine (CAP) in a phase I/II trial for patients with advanced solid tumors [33]. Twenty eight patients were in phase I, 14 patients were in phase II. The phase II regimen was VOR 300 mg daily plus CAP 1000 mg BID × 14 days per 21 day cycle. Preliminary results appeared to be encouraging.

In another phase I trial for patients with advanced solid tumors, VOR was combined with carboplatin (AUC = 6) and paclitaxel (200 mg/m^2^) [34]. VOR was given as 400 mg daily × 14 days or 300 mg BID × 7 days in a 3-week cycle. Twenty five of the 28 patients enrolled were evaluable. The DLT was emesis and neutropenia. 11 patients had PR, 7 patients had stable disease. Both VOR dosing schedule was well tolerated in combination.

Advanced mesothelioma progressed after first-line chemotherapy has poor prognosis. thirteen such patients were included in a single agent phase I trial [35]. Two patients had a partial response. This was considered to be promising for these poor-prognostic patients. A randomized phase III trial of oral VOR for patients with advanced mesothelioma are underway.

VOR 200 mg BID was also evaluated in a single agent phase II study for patients with recurrent/metastatic transitional cell carcinoma who failed platinum therapy [36]. Fourteen patients were included in the report at ASCO 2008 meeting. Two early on-study death was reported, and the study was closed to further accrual.

Single agent VOR at 400 mg daily had disappointing results in a phase II study for patients with platinum-resistant ovarian or primary peritoneal carcinoma [37]. VOR is also being studied in several phase I studies with a variety of regimens including gemcitabine/cisplatin for lung cancer, 13 cis-retinoic acid for children with solid tumors (Table 1 and 2) [38,39]. Final results are awaited. Single agent VOR for solid tumors and MM was overall disappointing in early clinical trials [22,37,40,41].

### New HDAC inhibitors

HDAC inhibitors generally consist of three parts in the chemical structure: (1) a zinc-chelating group; (2) a spacer group, which is generally hydrophobic; and (3) an "enzyme binding" group that confers specificity and is generally aromatic in character [42]. A spectrum of naturally occurring or synthetic HDAC inhibitors have been characterized for their antitumor activities in preclinical studies [43]. Six major classes of HDAC inhibitors have been defined on the chemical structures [44]. These include short-chain fatty acids (butyrate and valproic acid), hydroxamates (SAHA, trichostatin A, ITF2357, LBH589, oxamflatin, PCI-24781, and PXD101), benzamides (MS-275, CI-994, and MGCD-0103), cyclic tetrapeptides (depsipeptide, trapoxin A, and apicidin), electrophilic ketones (trifluoromethylketone), and miscellaneous (depudecin, SNDX-275, and isothiocyanates). In addition to vorinostat which has been approved for clinical treatment of advanced CTCL, there are at least 11 more HDAC inhibitors in various stages of clinical development (Table 3).

**Table 3:** New HDAC inhibitors in clinical trials

HDACI	Other agents	Dose & schedule	disease	Phase	Pts No	Outcome	Reference
CI-994	capecitabine	4–6 mg/m^2^	solid tumors	I	54	MTD:6 mg/m^2^	[49,50]
CI-994	gemcitabine	2–8 mg/m^2 ^po qd	solid tumors	I	20	MTD:6 mg/m^2^+ Gem1000 mg/m^2^	[47]
CI-994	paclitaxel carboplatin	4–6 mg/m^2^	solid tumors	I	30	MTD: 4 mg/m^2^	[48]
CI-994		8 mg/m^2 ^po qd	solid tumors	I/II	53	MTD:8 mg/m^2^	[46]
FK228	gemcitabine	7–12 mg/m^2^	Pancreatic, Solid tumors	I	33	MTD: 12 mg/m^2^+ Gem 800 mg/m^2^	[53]
FK228		1–9 mg/m^2^	thyroid, Solid tumors	I	26	MTD: 9 mg/m^2^	[54]
FK228		18 mg/m^2^i.v. d1 & d5, q3w	MDS, AML	II	12	CR: 8% SD: 50%	[55]
FK228		17.8 mg/m^2 ^d1 & d7, q 3w	lung cancer	II	19	No response	[56]
FK228		14 mg/m^2 ^i.v.d1, 8, 15 q4w	T-cell lymphoma	II	42	no myocardial damage	[58]
FK228		13 mg/m^2 ^i.v.d 1, 8, 15, q4w	Kidney CA	II	29	Insufficient activity (7% RR)	[57]
ITF2357		150 mg or 100 mgQ12 h 4 d	MM	II	16	PR: 6% SD: 31%	[61]
ITF2357		100 mg PO	Hodgkins	II	15	SD: 54%	[60]
LBH589	Docetaxel prednisone	15–20 mg po qod	CRPC	I	16	Oral study closed. IV form ongoing	[64]
LBH589		20 mg day 1, 3 5	CTCL	II	40	RR: 12%	[65]
LBH589		4.8–11.5 mg/m^2 ^IV	Hematological neoplasms	I	15	Study halted due to QTc prolongation	[63]
MGCD0103	gemcitabine	Phase I: 50–110 mg Phase II: 90 mg	solid tumors	I/II	29	MTD: 90 mg	[70]
MGCD0103		12.5–56 mg/m^2^/d	solid tumors	I	38	MTD: 45 mg/m^2^	[68]
MGCD0103		20–80 mg/m^2^	leukemia	I	29	MTD: 60 mg/m^2^	[69]
MGCD0103		85 mg, 110 mg	NHL	II	50	RR: 23%	[71]
MGCD0103		85 mg, 110 mg	HL	II	33	RR: 38%	[72]
MS-275		4–10 mg/m^2^	leukemia	I	38	MTD:8 mg/m^2^	[75]
MS-275		4–6 mg/m^2^	Solidtumors, lymphoma	I	27	MTD:6 mg/m^2^	[76]
MS-275		3–7 mg/m^2^	melanoma	II	28	SD: 21–29%	[77]
PCI-24781		2.0–2.4 mg/kg	solid tumors	I	15	MTD: pending	[49]
PBA		9–45 g/d	solid tumors	I	28	MTD:27 g/day	[82]
PBA		150–515 mg/kg/d	solid tumors	I	24	MTD:410 mg/kg	[81]
PBA		60 mg -360 mg/kg/d	solid tumors	I	21	MTD:300 mg/kg	[80]
PBA	5-FU	410 mg/kg/d	colorectal cancer	I	9	MTD: Not reported	[83]
PBA	azacitidine	200 mg/kg/d	AML MDS	II	10	RR:50%	[84]
PXD101		1000 mg/m^2^/d ×5	Ovarian ca	II	30	SD: 60%	[88]
PXD101		600, 900, 1000 mg/m^2^/day	Hematological neoplasms	I	16	MTD: 1000 mg/m^2^/day	[87]
PXD101		150–1200 mg/m^2^/day	solid tumors	I	46	MTD: 1000 mg/m^2^/day	[86]
VPA		30–120 mg kg(-1) day(-1) IV × 5	solid tumors	I	26	MTD: 60 mg/kg/day	[93]
VPA	ATRA	serum level 50–100 mcg/ml	AML	II	58	RR: 5% (not active)	[90]
VPA	ATRA	5–10 mg/kg	AML	I	26	Insufficient activity	[91]
VPA		20 – 40 mg/kg/d × 5	Cervical ca	I	12	MTD: Not reported	[92]

HDAC: histone deacetylase inhibitor; CR: complete responses; PR: partial response; SD: stable disease. MTD: maximal tolerated dose; RR: response rate

#### 1. CI-994 (N-acetyldinaline, [4-(acetylamino)-N-(2-amino-phenyl) benzamide])

CI-994 is an orally active HDAC inhibitor that belongs to the benzamide class [45]. A phase I/II study was carried out in patients with solid tumors. Fifty three patients received CI-994 orally for 2–10 weeks [46]. Thrombocytopenia was the DLT. The MTD was 8 mg/m^2^/day for 8 weeks. One refractory lung cancer patient had PR for over 2 years, 3 additional patients had stable disease.

CI-994 was investigated in combination with gemcitabine in a phase I trial for solid tumors [47]. Twenty patients were treated with gemcitabine (1000 mg/m^2 ^on days 1, 8, 15). CI-994 was given orally in a dose-escalating schedule from 2–8 mg/m^2^/day in a 21-day cycle. The DLT was thrombocytopenia, and the MTD was 6 mg/m^2 ^for combination with gemcitabine. CI-994 was also studied in combination with paclitaxel and carboplatin in a phase I trial in patients with advanced solid tumors [48]. CI-994 dose ranged 4–6 mg/m^2 ^for one or two weeks. Thirty patients were enrolled. The MTD was 4 mg/m^2 ^for 7 days for the combination regimen. CI-994 was evaluated in another phase I trial in combination with capecitabine [49,50]. Fifty four patients with advanced solid tumors were enrolled. CI-994 was given in a dose-escalating schedule from 4–6 mg/m^2 ^daily. The DLT was thrombocytopenia. The MTD was 6 mg/m^2 ^daily for two weeks in a 21-day cycle in combination with capecitabine.

#### 2. FK228 (FR901228, depsipeptide, romidepsin)

FK 228 is a potent bicyclic depsipeptide and a novel HDAC inhibitor [51,52].

FK228 was studied in combination with gemcitabine in a phase I trial for patients with advanced solid tumors [53]. Thirty three patients were included in the report. Non-hematologic toxicities have been mild to moderate nausea, vomiting, and fatigue. The recommended phase II dose schedule is FK228 (romidepsin) 12 mg/m^2 ^and gemcitabine 800 mg/m^2 ^every other week.

HDAC inhibitors restore expression of the sodium iodine symporter in refractory cells and sensitivity to RAI in vitro. A phase I trial was conducted for patients with thyroid and other advanced cancers using FK228 (romidepsin) on days 1, 3, 5 [54]. Twenty six patients were enrolled. Severe adverse events were hematologic and GI toxicities. The MTD is 9 mg/m^2^. Histone acetylation was shown to have a greater than 2-fold increase. This study was planned to focus exclusively on non-medullary thyroid cancer.

FK228 (depsipeptide) was also evaluated in a phase II study for patients with high risk MDS and AML [55]. FK228 was given on day 1 and day 8 to 12 patients at 18 mg/m^2 ^over a 4-hour infusion every 3 weeks. There was one CR, six stable diseases. Histone H3 and H4 acetylation was seen, but there was no consistent changes.

Another phase II trial of FK228 was done in patients with refractory lung cancer [56]. Nineteen patients were treated on day 1 and 7 every 3 weeks at a dose of 17.8 mg/m^2^. Hematologic toxicity was dose-limiting in one patient, no objective responses were observed in this single agent study.

In another single-agent phase II trial, FK228 was given to patients with refractory metastatic renal cell carcinoma at 13 mg/m^2 ^on days 1, 8, and 15 of a 28-day cycle [57]. Twenty nine patients were enrolled. Four patients had severe cardiac toxicity with one sudden death. There was only a 7% overall response rate. The study was closed due to insufficient efficacy. In a separate study with detailed monitoring of cardiac toxicities in 42 patients with T-cell lymphoma, Fk228 was given at 14 mg/m^2 ^on days 1, 8, and 15 of a 28-day cycle [58]. FK228 was not found to be associated with myocardial damage or decrease in cardiac function even though EKG changes with T-wave flattening or ST-segment depression were observed. The cardiac toxicities are believed to be a class effect of HDAC inhibitors.

#### 3. ITF2357

ITF2357 is an orally effective member of hydroxamic family of HDAC inhibitors and can reduce production of inflammatory cytokines [59]. ITF2357 was investigated in an Italian phase II trial on patients with heavily pretreated refractory Hodgkin's disease [60]. ITF2357 was given at 100 mg PO daily. Fifteen patients were enrolled, 13 were evaluable for responses. Stable diseases were seen in seven (54%) patients. 20% of the patients had QTc interval prolongation prompting transient drug discontinuation. Overall it was reported to be well tolerated.

A phase II study reported at ASH 2007 annual meeting administered 150 mg or 100 mg of ITF2357 orally every 12 hours for four consecutive days followed by a 3 day rest every week of a 28-day cycle [61]. Sixteen patients with refractory MM were treated. The most common grade 3–4 toxicities were GI side effects, neutropenia, and thrombocytopenia. Three patients had abnormal EKG changes. One patient achieved partial response, and five others had stable diseases.

#### 4. LBH589 (panobinostat)

LBH589 is a novel pan-HDAC inhibitor. Treatment with LBH589 not only has been shown to induce acetylation of histones, induction of p21, cell cycle growth arrest, and apoptosis but also has been demonstrated to induce acetylation of HSP90 [62].

LBH589 IV formulation was investigated in a phase I trial for patients with refractory hematological malignancies [63]. LBH589 as a single agent was administered as a 30-minute i.v. infusion once daily on days 1 to 7 of a 21-day cycle. The doses ranged from 4.8 mg/m^2 ^to 14 mg/m^2^. 15 patients were enrolled. The DLT was QTc prolongation at 14 mg/m^2^. A significant increase in acetylation of the H2B and H3 histones in the leukemic blast (CD34^+^) cells is consistent with LBH589 reaching its target. The study was halted due to safety concerns about QTc prolongation.

Oral LBH589 was studied alone and in combination with docetaxel and prednisone in castration-resistant prostate cancer [64]. 20 mg of oral LBH589 was administered on days 1, 3 and 5 for 2-week on and 1-week off schedule for LBH589 alone arm, 15 mg of LBH589 was given following the same schedule in the combination arm. Eight patients were enrolled into each arm. There was no apparent synergistic effect in the combination arm. Three patients achieved PR as the best responses. This study was closed and further clinical trials are being focused on IV formulation which produces higher peak concentration with comparable toxicity profile.

LBH589 was tested in a phase II trial in patients with CTCL [65]. LBH589 was administered orally at 20 mg on days 1, 3, and 5 weekly until disease progression. Patients with cardiovascular abnormalities or QTc>450 msec were excluded. Intensive ECG monitoring was performed. 40 patients have been enrolled in the report. Five patients achieved skin response including one complete skin response. Another patient with PD improved to PR after initial flaring of disease. There was no QTc>500 ms observed.

#### 5. MGCD0103

MGCD0103 is a selective orally available benzamide HDAC inhibitor that targets HDAC 1, 2, 3 (class 1) and 11 (class 4) [66,67]. It avoids the class 2 enzymes.

MGCD0103 was studied in a phase I trial for patients with advanced solid tumors. It was administered orally three-times-per-week for 2 of every 3 weeks [68]. The dose ranges were from 12.5 mg to 56 mg/m^2 ^in 38 patients over 99 cycles. The DLT included fatigue, nausea, vomiting, and diarrhea. The recommended phase II dose was 45 mg/m^2^/day. There was inhibition of HDAC activity and induction of acetylation of H3 histones by MGCD0103.

A separate phase 1 trial of oral MGCD0103 was conducted in patients with leukemia and myelodysplastic syndromes [69]. MGCD0103 was administered orally 3 times weekly without interruption. Twenty-nine patients with a median age of 62 years (32–84 years) were enrolled at dose levels of 20–80 mg/m^2^. The DLT were similar to those reported from the previous study [68]. The maximum tolerated dose was determined to be 60 mg/m^2^. Three patients achieved a complete bone marrow response.

MGCD0103 was also evaluated in a phase I/II trial in combination with gemcitabine in patients with solid tumors [70]. Twenty-nine patients were enrolled (25 in phase I, 4 in phase II). Dose levels of MGCD0103 ranged between 50 and 110 mg. The MTD and recommended phase II dose was 90 mg. 2 of 5 pancreatic cancer patients achieved PR. Phase II at 90 mg MGCD0103 3 ×/week plus gemcitabine 100 mg/m^2 ^weekly × 3 per 4-week cycle is ongoing for pancreatic cancer patients.

A phase II trial of oral MGCD0103 was conducted in patients with refractory large B-cell (DLBCL) or follicular lymphoma [71]. Among 50 patients enrolled, 32 patients received 110 mg three times per week. The dose was reduced to 85 mg 3 ×/week afterwards. 1 CR and 3 PRs with a response rate of 23.5% were achieved in 17 patients with DLBCL. Inhibition of HDAC activity was seen in 13 of 18 patients evaluated. In a separate phase II study, patients with refractory Hodgkin lymphoma were enrolled for treatment with MGCD0103 [72]. Twenty three patients received 110 mg, 10 patients had 85 mg 3 ×/week in 4-week cycles. Most patients had failed prior autologous transplants. Among the 110 mg cohorts, 2 patients achieved CR, 6 achieved PR, for an overall response rate of 38%. The median time to response was 2 cycles. The 85 mg dose was better tolerated and further study at this dose level is ongoing.

#### 6. MS-275 (MS-27–275; *N*-(2-aminophenyl)-4-[*N*-(pyridin-3-yl-methoxycarbonyl) aminomethyl] benzamide)

MS-275 is a novel synthetic benzamide derivative that has been shown to inhibit HDAC activity [73,74]. A phase I dose-escalating study has been completed in patients with advanced acute leukemia [75]. Thirty eight patients were enrolled. The first 13 patients were treated with MS-275 initially once weekly × 2, repeated every 4 weeks from 4 to 8 mg/m^2^. The rest of the patients were treated once weekly × 4, repeated every 6 weeks from 8 to 10 mg/m^2^. The MTD was 8 mg/m^2 ^weekly for 4 weeks in 6-week cycle. The DLTs included infections and neurologic toxicity manifesting as unsteady gait and somnolence. MS-275 induced H3 and H4 acetylation.

MS-275 was also investigated in patients with solid tumors in a phase I trial [76]. Twenty seven patients with advanced solid malignancies and lymphomas were treated on three dose schedules. MS-275 is well tolerated at doses up to 6 mg/m^2 ^every other week or 4 mg/m^2 ^weekly for 3 weeks. The DLTs were hypophosphatemia and asthenia on the weekly and twice-weekly dosing schedules; there was no dose-limiting toxicity on the every other week schedule. Four mg/m^2 ^given weekly for 3 weeks every 28 days were recommended for phase II study.

A phase II trial was done on patients with refractory metastatic melanoma [77]. Twenty eight patients were randomized to receive MS-275 3 mg biweekly (days 1+15, arm A) or 7 mg weekly (days 1+8+15, arm B), in 4-week cycles. Nausea and hypophosphatemia were the most common toxicities. No objective response was reported. Stable disease was observed (29% in Arm A, 21% in Arm B). Single agent MS-275 appears to be ineffective in this population of patients.

#### 7. PCI-24781 (CRA-024781)

PCI-24781 is a novel, broad spectrum hydroxamate-based inhibitor of HDAC that shows preclinical antitumor activity [78]. A phase I study was done in patients with solid tumors [49]. 15 patients were reported at the ASCO 2008 annual meeting. Intravenous and oral forms are both studied. Tubulin and histone acetylation were measured in peripheral blood mononuclear cells. Hematologic and GI toxicities were observed, and 1 patient had EKG changes. Acetylation levels increased at 1.5 hour post dose and sustained through 4 hours in all patients and up to 24 hours in 60% of the patients. PCI-24781 was well tolerated following IV administration. Further study of oral formulation is ongoing.

#### 8. Phenylbutyrate

Phenylbutyrate (PBA) is an aromatic short-chain fatty acid that has activity in HDAC inhibition [79]. Phase I clinical studies have been done [80-82]. Oral PBA was evaluated in a phase I trial [82]. Twenty eight patients with refractory solid tumors were included. Five dose levels (9 gm/day to 36 gm/day in three divided doses) were studied. The DLTs were nausea, vomiting and hypocalcemia at 36 gm/day. 27 gm/day was the recommended phase II dose. PBA was administered intravenously as 120-hour infusion in 24 patients with solid tumors in a separate phase I trial [81]. Six dose levels were studied (150 mg to 515 mg/kg/day in 21-day cycles). The DLTs were mainly neurological, such as somnolence and confusion. The MTD was 410 mg/kg/day for 5 days. Another phase I trial evaluated twice-daily PBA infusions for two consecutive weeks every month at five doses levels (60 mg -360 mg/kg/day) in patients with advanced solid tumors [80]. The MTD was 300 mg/kg/day.

PBA was also studied in combination with 5-fluouracil (FU) in a phase I trial. FU (24-hour continuous intravenous infusion (CIV)) with dose escalation (2–2.3 g/m^2^), in combination with PB (120 hour CIV at fixed dose 410 mg/kg/d × 5) was administered weekly in patients with advanced colorectal cancer [83]. Nine patients were enrolled. MTD has not been reached at the time of report. PBA was also combined with azacitidine in a phase II trial for patients with AML and MDS [84] (see above under section of azacitidine).

#### 9. PXD101 (belinostat)

PXD101 is a novel hydroxamate-type HDAC inhibitor [43,85]. A phase I trial of PXD101 was performed on patients with advanced solid tumors [86]. Forty six patients were enrolled. 6 dose levels were tested. The DLT were grade 3 fatigue. The MTD was determined to be 1000 mg/m^2 ^IV infusion over 30 minutes daily for 5 days per 21-day cycle. Histone H4 hyperacetylation was observed after each infusion and was sustained for 4 to 24 hours in a dose-dependent manner. Of the patients treated at the MTD, 50% achieved stable disease.

Another phase I dose-finding study was done in patients with advanced hematological malignancies [87]. Sixteen patients were enrolled. Four dose levels were included. One patient developed drug-related grade 3 toxicities, including fatigue and neurological symptoms. The MTD was same as above and was to be used for phase II studies.

One phase II study of PXD101 was reported at 2008 ASCO annual meeting [88]. In this study, 30 patients with metastatic or recurrent and refractory ovarian cancer were enrolled. Eighteen out of the 30 patients had stable diseases. The study appears to be promising, and recruitment was still ongoing.

#### 10. Valproic acid

Valproic acid (VPA, 2-propylpentanoic acid) is a well established drug for the therapy of epilepsy. It is teratogenic when administered during early pregnancy and can induce birth defects such as neural tube closure defects and other malformations. The well-tolerated antiepileptic drug was found to be a powerful HDAC inhibitor. VPA induces differentiation of carcinoma cells, transformed hematopoietic progenitor cells and leukemic blasts from acute myeloid leukemia patients [89].

VPA was studied in combination with All-trans retinoid acid (ATRA) in patients with AML who were not candidates for intensive chemotherapy [90]. Fifty eight patients were enrolled. Forty patients received the combination therapy. There was only 5% response rate for this group of patients with no CR observed. Another trial of VPA (5–10 mg/kg) plus ATRA (45 mg/m^2^) was done on 26 patients with poor-risk AML [91]. No patients achieved CR. These studies suggest that additional trials are needed to clearly define the activity of VPA in poor-risk AML patients.

A phase I trial of single agent VPA was reported in patients with newly diagnosed cervical cancer [92]. Twelve patients were included. VPA doses ranged from 20 mg/kg to 40 mg/kg daily for 5 days. The most common side effect was depressed level of consciousness which was not severe. Tumor HDAC activity decreased in 8 patients. However, there was no correlation between H3 and H4 hyperacetylation with serum levels of VPA.

VPA was studied for IV administration in a phase I trial for patients with advanced cancer [93]. Twenty six patients were enrolled. VPA was administered as a 1-hour infusion daily for 5 consecutive days in a 21-day cycle with doses ranging between 30 mg/kg/day and 12 mg/kg/day. The MTD was 60 mg/kg/day. The DLT was grade 3 or 4 neurological impairment occurring in 8 out of 26 patients.

#### 11. Other HDAC inhibitors in early stage of clinical development (Isothiocyanates, NVP-LAQ824, SNDX-275)

Isothiocyanates (ITC) can be found as thioglucoside conjugates, i.e. glucosinolates, in a wide variety of cruciferous vegetables including broccoli, cabbages, watercress, and Brussel's sprouts, etc. A phase I study of glucosinolate and ITCs (sulforaphane) were done in healthy volunteers [94]. The excretion of a metabolite, dithiocarbamates, was measured. No clinically significant toxicities were observed. Sulforaphane and phenylhexyl isothiocyanate (PHI) are among the synthetic isothiocyanates that are shown to be HDAC inhibitors and have antitumor activities in vitro and in vivo [95-100]. PHI was found recently to have dual epigenetic effects as both HDAC inhibitor and hypomethylating agent [97]. Clinical development of ITCs is underway.

NVP-LAQ824 ((2E)-N-hydroxy-3-[4-[[(2-hydroxyethyl)[2-(1H-indol-3-yl)ethyl]amino]methyl]phenyl]-2-propenamide) is a structurally novel hydroxamate derivative of HDAC inhibitors [101,102]. It has broad antitumor activity in preclinical studies [103-106]. Clinical trials in human are currently underway.

SNDX-275 is another novel HDAC inhibitor and is currently undergoing a phase I trial in combination with azaciditine (see above under section of azacitidine).

There are more structurally novel HDAC inhibitors which have been shown to have preclinical antitumor activities [43]. Clinical developments are yet to be done.

## Conclusion

Vorinostat is the first HDAC inhibitor that has been approved for treatment of CTCL. More than 11 HDAC inhibitors are in various stages of clinical development. HDAC inhibitors may have more potential in the combination therapy of a wide range of malignancies. Combination of novel epigenetic agents, including hypomethylating agents and HDAC inhibitors, and chemotherapeutic agents are being extensively investigated for clinical treatment of malignant disorders. Results from the clinical trials are eagerly awaited and being closed watched.

## Competing interests

The authors declare that they have no competing interests.

## Authors' contributions

SC and DL are involved in concept design. All authors participated in data collection, drafting and critically revising the manuscript.
